# Supplementary material for: Are carbon emissions trading and green financial instruments synergistic? -Comprehensive quantitative research based on content analysis
Source: PLoS One. 2024 Mar 7;19(3):e0298601. doi: 10.1371/journal.pone.0298601 (PMC10919601; doi:10.1371/journal.pone.0298601)
Supplement: S1 Data — (ZIP) [file pone.0298601.s001.zip › Data/地级市Z_F工作报告.zip!/╔╜╬≈/┬└┴║╩╨/└┤╫╘╣1⁄2╓┌║┼í╛┬φ┐╦╩2╛▌═°í┐_2018.docx]

　　现在，我代表市人民政府向大会报告工作，请予审议，并请市政协委员和列席人员提出意见。

　　一、2017年工作回顾

　　2017年是吕梁发展进程中极不平凡的一年，是具有重大里程碑意义的一年。6月21日习总书记亲临山西视察，第一站就来到吕梁，充分肯定了吕梁精神。党和人民的领袖对老区人民的亲切关怀，使全市上下备受鼓舞、倍感振奋、干劲倍增。我们牢记总书记嘱托，在市委的坚强领导下，坚持以习近平新时代中国特色社会主义思想为指导，全面贯彻党的十九大精神和习总书记视察山西重要讲话精神，不断增强政治意识、大局意识、核心意识、看齐意识，深入贯彻落实新发展理念，扎实推进脱贫攻坚、转型升级、全面从严治党“三件大事”，吕梁同全省一样政治生态由“乱”转“治”、发展由“疲”转“兴”，脱贫攻坚再战再胜，经济社会发展取得了历史性成就、发生了历史性变革。

　　全年地区生产总值完成1310.3亿元，增长9.2%；规模以上工业增加值完成878.4亿元，增长11.3%；一般公共预算收入完成138.8亿元，增长54.9%；企业利润总额实现195.9亿元，增长44.6倍。4项主要经济指标增速从2015年全省末位，2016年由负转正进入中游，2017年上升为第一位，实现了年初提出的“保中游、争上游”的目标。固定资产投资增长6.2%，社会消费品零售总额增长6.4%，城镇、农村居民人均可支配收入分别增长6.3%、7.7%，达到25704元、8232元。市区空气质量优良天数排名全省第二。以全国林业扶贫现场会等10多个国家和省现场会为标志，生态脱贫、光伏扶贫等一批重点工作进入全省、全国“第一方阵”，广大干部群众干事创业氛围日益浓厚，全市内生动力和外部形象都发生了深刻变化。

　　一年来，我们主要做了以下工作。

　　（一）坚持以脱贫攻坚统揽经济社会发展全局，聚焦深度贫困，脱贫攻坚再战再胜。压茬推进“春季行动”“夏季攻势”“秋季会战”“冬季对标”，全年445个贫困村退出、14.8万贫困人口减贫，中阳、柳林2个贫困县摘帽，孝义、汾阳、文水3个县整体脱贫。打响具有吕梁特色的生态脱贫、护工护理培训和光伏扶贫“三张品牌”，在生态建设战场打赢增绿增收两场战役的做法得到习总书记充分肯定，国家林业局长、扶贫办主任及全国20多个省区林业厅长和扶贫办主任在离石、柳林、临县观摩了现场；“吕梁山护工”品牌叫响全国，央视《朝闻天下》栏目连续8期报道；光伏扶贫走在全国前列。部分省区领导观摩了交城易地扶贫搬迁安置点，时任副总理汪洋同志亲临现场给予高度评价。大力实施特色产业扶贫，带动33.1万贫困人口增收。加大金融扶贫力度，风险补偿产业扶贫贷款模式全国推广。出台教育扶贫“5+5”资助政策，资助贫困学生20.9万人次；贫困人口医疗费用报销率达87.34%，全省排名第二；13个县市区农村低保标准与扶贫标准实现“两线合一”，低保标准全部高于扶贫标准，脱贫攻坚“三五工程”方略开始落地生根。

　　（二）坚持加快转型发展不动摇，大力实施产业发展战略，产业集群化发展水平有效提升。抓住国发42号文件政策机遇，打出转型综改“组合拳”，推进九大产业转型发展。铝工业逐步发展壮大，局域电网初步建成试用，全省最大的合金铝生产线中润一期50万吨项目具备投产条件。煤焦产业向化的方向延伸，鹏飞集团与北京三聚环保公司合作的30万吨甲醇联产2亿立方LNG项目建成投产，金达、金岩分别与宝钢、山东科瑞合作，上马了煤系针状焦、甲醇联产LNG等传统产业升级项目。白酒产业加快发展，产量和产值均增长40%以上，我市被评为“世界十大烈酒产区”之一。文化旅游产业提档升级，开展“乘汾酒号列车，赏吕梁山风光”“吕梁—一个令人向往的地方”旅游季活动，全年接待游客人数、旅游总收入增幅均超过30%，我市荣获2017亚洲旅游产业年会“最受欢迎旅游目的地”奖。大数据产业迅猛发展，依托华为公司引进软通动力等20家企业。规上工业增加值中，非煤产业占比由2013年的35.5%上升到49%，其中铝工业、白酒产业分别达16.6%、6.8%，战略性新兴产业和高技术产业增加值分别增长16.9%、14.3%，产业转型步伐不断加快，优势产业做强、新兴产业做大的格局开始显现。

　　（三）坚持全面深化改革开放，着力破除体制机制障碍，发展动能加速转换。认真落实“三去一降一补”重点任务，核减煤炭产能20万吨，减量置换234.6万吨；加大房地产去库存力度，全市商品房待售面积、库存消化周期实现“双下降”；多措并举降低企业负债率和实体经济成本，规上企业每百元主营业务收入成本减少5.79元。成立9户售电公司，38户企业参与电力直接交易，节约成本1.5亿元。加快开发区“整合改制扩区调规”，基本完成“三制”改革，省级开发区数量达到5个。19个景区完成体制机制改革，实现管理权和经营权“两权分离”。87户国有企业完成公司制改革，离柳焦煤集团等18户试点企业改革迈出坚实步伐。举办“数谷吕梁·智赢未来”大数据产业推介会、世界酒文化博览会、第四届全国煤炭深加工及焦化产业绿色发展交流会、铝工业发展论坛。搭建的这四大招商和转型发展大平台，吸引了12位两院院士、100多位专家学者及5000多领军企业和人员参加，引进转型项目30多个，扩大了吕梁影响、塑造了吕梁新形象。大力实施“百千万人才工程”和“百校千人计划”，与北航、北理工等9所重点高校（单位）签订战略合作协议，47位院士专家被聘为吕梁转型发展顾问。建立博士后工作站、技术研发平台12个。吕梁市推广PPP模式和孝义公立医院改革受到国务院通报表扬。特别是涉及1.7万职工、1820户普通债权人、586亿元债务的联盛集团依法破产重整，初步实现“平稳重整、涅槃重生”目标，成为全国化解金融和社会风险的典型案例，受到省委省政府和中国银监会的充分肯定。

　　（四）坚持实施新型城镇化发展战略，狠抓基础设施建设，城乡面貌持续改善。大力推进“双十”市政重点工程建设。新区以11.5公里吕梁大道为主干的两纵十六横路网基本建成；以两带三湖为主的水面面积达到327亩、绿化面积达到2326亩，宜居宜业生态环境初步形成；吕梁火车站站前广场、盛地小学投入使用，水电气热讯等市政设施建成完善；社会关注的两县区16个小区69栋安置楼11369套安置房建成，4000多户群众拿到新房钥匙，进一步加快推进新区建设成为广大干部群众的期盼和共识。主城区提质改造步伐加快，投资9亿多元实施城区集中供热项目，供热面积从1477万平米增加到近2000万平米，热源点由原来的3个增加到5个，惠及2万多户群众；雨污分流管网改造工程完成；城南传统工业主题公园一期、兴隆、永宁小游园等8项宜居环境改善项目基本建成或投入使用。启动“五十百千”创建活动，孝义创建全国文明城市，汾阳、岚县创建省级文明城市，石楼创建省级卫生城市，汾阳贾家庄、离石信义创建国家特色小城镇，孝义梧桐、临县碛口、柳林孟门创建省级特色小城镇。开工建设静兴、祁离2条高速和沿黄扶贫旅游公路一期工程，新改建农村公路1210公里。榆横-潍坊、上海庙-山东过境输电线路全面架通。中部引黄隧洞连通工程和柏叶口龙门供水工程建成投运。

　　（五）坚持以人民为中心的发展思想，着力保障和改善民生，人民获得感不断增强。扎实推进习总书记提出的12项民生实事、省政府6件实事和我市6件便民实事。全年民生支出259.9亿元，占公共财政支出的82.49%。提前一年完成“义务教育基本均衡评估认定”。孝义教育改革走在全国前列，全国义务教育学校管理标准实施部署会在孝义召开。高考二本B类以上达线人数超过1.4万，清华、北大录取18人，实现历史性突破。大力推进医疗、医保、医药“三医联动”，孝义、岚县、交口经验做法被央视报道，全省医改工作现场会在柳林召开。开展大气、水、土壤污染防治三大战役，扎实推进中央、省环保督察发现问题整改工作，投资近100亿元实施50项重点环保基础设施工程，全市空气质量和重点河流水环境质量大幅改善，17个重点河流水质监测断面达到考核要求。毫不松懈抓好安全工作，安全生产形势总体平稳，被省政府考核为优秀。深入开展扫黑除恶专项斗争，刑事案件和治安案件分别下降22.43%、13.32%，人民群众安全感有所提升。

　　（六）坚持不懈推进党风廉政建设，依法全面履行职能，政府效能有效提升。切实加强政府系统全面从严治党，深入推进反腐败斗争。扎实推进法治政府建设，严格执行市人大及其常委会的决议决定，主动接受人大、政协监督，人大代表建议和政协委员提案办结率达100％。建成市县乡三级“13710”电子督办平台，全年督办重点工作233件，政府执行力持续提升。建立新区建设、集中供热、扶贫等重点工作微信平台，实行“3个24小时直通车”制度，及时协调解决各类问题1000余个，实现政府服务高效率快作为。深化“放管服效”改革，取消和下放审批事项145项。扎实开展干部入企服务常态化等十大专项行动，在孝义试点推行企业投资项目承诺制。支持各民主党派、人民团体工作，国防动员、人民防空、民兵预备役建设进一步加强，粮食供销、外事侨务、文物保护、防震减灾、民族宗教、档案方志、应急管理、对台、气象等工作取得新进步。

　　各位代表，回顾过去的一年，我们取得了来之不易、振奋人心的成绩，根本在于有习总书记这一全党拥护、人民爱戴的领袖为我们领航掌舵，在于以习近平同志为核心的党中央的英明领导，在于有习近平新时代中国特色社会主义思想科学指引；这些成绩的取得是省委、省政府亲切关怀、坚强领导的结果，是市委统揽全局、正确领导的结果，是市人大有效监督、市政协大力支持的结果，是全市人民勠力同心、砥砺奋进的结果。在此，我谨代表市人民政府，向全市人民、全体人大代表和政协委员，向各民主党派、人民团体和各界人士，向驻吕解放军、武警官兵和公安干警，向所有关心支持吕梁改革发展的朋友们，表示衷心的感谢和崇高的敬意！

　　各位代表，安不忘危、兴不忘忧。我们清醒地认识到，发展不平衡不充分的问题在我市表现十分突出，政府工作还有不少问题。煤焦冶电传统产业占比较高，战略性新兴产业和高技术产业发展不足，工业转型类投资增长乏力；城乡居民收入水平滞后于经济发展水平，全省10个深度贫困县我市就有3个，增收任务仍然十分艰巨；城镇化水平不高，民生领域还有不少短板，市区学校、医院建设不能满足人口增长需要；生态环境脆弱，环保基础设施欠账较多，采暖期大气污染严重，去年市区尽管新增供热面积500万平米，但因政策因素晋能大土河两台供热发电机组仅批复一台，部分区域供热温度不高不稳定；金融和安全领域风险隐患不容忽视，社会治安形势依然复杂；一些干部干事创业精气神不足，不敢为、不会为、不善为，庸政懒政怠政现象仍然存在，等等。对此，我们一定以不畏艰难、坚韧不拔的勇气和意志，以持之以恒、奋发有为的精神和毅力，不懈怠，不松劲，全心全意、尽心竭力认真加以解决，不辜负人民的重托！

　　二、2018年工作安排

　　2018年是全面贯彻党的十九大精神的开局之年，是改革开放40周年，是决胜全面小康、实施“十三五”规划承上启下的关键一年。做好政府工作，要全面贯彻党的十九大精神，以习近平新时代中国特色社会主义思想为指导，坚持稳中求进工作总基调，坚持新发展理念，紧扣社会主要矛盾变化，按照高质量发展的要求，统筹推进“五位一体”总体布局和协调推进“四个全面”战略布局，坚持以脱贫攻坚统揽经济社会发展全局，坚持把深化供改与深化综改结合起来，围绕建设“示范区”“排头兵”“新高地”三大目标，聚焦防范化解重大风险、精准脱贫、污染防治“三大攻坚战”，实施“十大举措”，把握“六个结合”，统筹推进稳增长、促改革、调结构、惠民生、防风险各项工作，大力推进改革开放，推动质量变革、效率变革、动力变革，促进经济社会持续健康发展。

　　主要预期目标是：地区生产总值增长7%左右；一般公共预算收入增长8.5%；固定资产投资增长9%；社会消费品零售总额增长7%；城镇居民和农村居民人均可支配收入分别增长6.5%、6.6%。城镇调查失业率和登记失业率分别控制在6%、4.2%以内。

　　约束性指标是：严格执行能源消耗总量和强度“双控”制度，万元GDP综合能耗下降率、万元GDP二氧化碳排放量、万元GDP用水量降幅等减排指标，完成省定任务。市区空气质量持续保持在全省重点城市前列。

　　2018年总体要求和主要指标设定，坚持高质量发展要求，充分考虑吕梁经济恢复性增长的实际，既实现量的快速增长，为转型发展和脱贫攻坚积蓄力量；又实现质的大幅提高，为产业转型和深化改革留出空间，确保速度、效益、结构、动能四个维度的同步协调、相互支撑。争取各项主要经济指标在去年“保中游、争上游”的基础上，实现“双保双创”目标,即：主要经济指标增速和总量在全省保上游，脱贫攻坚、产业转型在全省创一流。我们要“干”字当头，以对事业饱满的热情、对工作旺盛的激情，撸起袖子，甩开膀子，干出推动高质量发展的“加速度”，干出攻坚深度贫困的“绣花功”！

　　围绕以上目标，重点抓好以下工作：

　　（一）攻坚深度贫困堡垒，实现脱贫攻坚连战连胜。

　　今年的脱贫任务更重，脱贫难度也更大，年内要确保354个贫困村退出、12.6万人减贫，方山、岚县、交城、交口、离石5个贫困县（区）摘帽脱贫。我们将全面贯彻习总书记扶贫开发重要战略思想，按照市委“脱贫攻坚要跻身全省全国第一方阵”的要求，突出“六个坚持”、把握“五个转变”，以“打不赢脱贫攻坚战，就对不起这块红色土地”的坚定决心，举全市之力再打一个漂亮仗。

　　提升生态脱贫、护工护理培训、光伏扶贫“三大品牌”。坚定不移推进生态脱贫。认真践行总书记“绿水青山就是金山银山”的理念，以规模化、合作化方式集中实施“三个100万亩工程”，即荒山造林100万亩、退耕还林100万亩、经济林提质增效100万亩，全面启动国家储备林项目，加快推进森林城市建设。实施造林务工、退耕奖补、管护就业、经济林提质增效、苗圃育苗“五大项目”。进一步加大护工护理培训力度。紧紧抓住贫困人口参训、培训质量、转移就业、跟踪服务四个环节，举办“吕梁山护工就业推介会”，筹建“吕梁山护工”服务部，打造一站式“家政服务超市”，进一步打响“吕梁山护工”品牌。年内完成1.25万人的培训任务，其中贫困人口培训率力争达到60%以上、就业率达60%以上。加强与中铝、山西焦煤等大型企业合作，抓好吕梁山技工、吕梁山工匠等全民技能提升培训，实现持证上岗，不断提高贫困群众就业增收能力。扎实推进光伏扶贫。全面铺开总规模416.68MW的260个村级电站、25个联村电站、5座集中式电站建设，力争10月底前全部并网发电。进一步完善收益分配办法，保障深度贫困户优先受益，带动1124个贫困村集体经济收入稳定达到5万元以上。

　　坚决啃下易地扶贫搬迁“硬骨头”。以深度贫困村为重点，围绕解决“人钱地房树村稳”等问题，“六环联动”有序推进，确保2017年搬迁人口年底前全部入住，剩余4.33万人的搬迁任务年底前全部竣工。用足用好城乡建设用地增减挂钩等政策，整村拆除复垦自然村510个。大力发展后续配套产业，让贫困群众搬得出、稳得住、逐步能致富。

　　大力推进企业产业扶贫。广泛动员央企、省属企业和民营企业等，本着自愿、双赢、互补的原则，启动实施绿化一座山、治理一条沟、扶持一个主导产业、安排一批劳动力、救助一批特困群众等“八个一”为主要内容的多种途径、多种方式的企业帮扶。探索资源型企业以工补农，助力产业脱贫的新途径。总结推广离石泰化“三转三化”、中阳中钢帮助搬迁群众装修安置房、汾阳汾州香“政府+研究所+公司+农户”、兴县山花烂漫“公司+农户”等模式，增加贫困群众收入。

　　提高社会保障水平。加大健康扶贫力度。持续开展“三个一批”医疗救治行动，大力推行贫困人口住院先诊疗后付费“一站式”服务、健康扶贫“双签约”等机制，确保农村贫困人口住院总费用实际报销比例达到90%，健康扶贫政策真正落实到位。持续落实教育扶贫政策。建立教育扶贫个人资金账户，继续对贫困县农村幼儿园、义务教育和普通高中、中高等职业学校以及二本B类以上高校贫困生进行资助。对贫困县农村寄宿制学校所有寄宿生、非贫困县农村寄宿制学校的建档立卡贫困家庭子女和特殊教育学校寄宿生给予免费就餐；租房居住不在学校就餐的小学生，给予生活补助，确保建档立卡贫困家庭每一个学子真正享受到资助政策。提高兜底扶贫标准，摸清找准低保对象，稳步提高低保标准，持续推进“两线合一”，确保农村低保标准高于国家扶贫标准。加快养老保险参保人群全覆盖步伐，城乡居民基础养老金最低标准提高到103元，进一步增加60周岁以上贫困人口收入。实施贫困村提升工程，加强贫困乡村公路、安全饮水、电网、物流、互联网等基础设施建设，村级公路通畅率达到100%，年内脱贫摘帽县完成危房改造8182户，解决364个退出贫困村饮水安全问题。对整村搬迁以外退出贫困的农村，实施整村提升工程，确保贫困地区基本公共服务主要指标接近全省平均水平。

　　积极创新帮扶机制。凝聚帮扶合力。进一步完善驻村帮扶工作队“三四五”管理制度，继续加大工作经费支持，压实帮扶责任，发挥好“三支力量”帮扶作用；加大县际结对帮扶力度，充分发挥民主党派、工商联、群团组织等桥梁纽带作用，鼓励引导各类社会组织、企业和个人开展向贫困地区、贫困群众献爱心活动，为帮扶县提供人力、物力、财力、智力支持。创新投入机制。继续加大涉农资金整合使用力度，确保应整合和实际整合规模、支付进度均达到90%以上。创新财政投入方式，利用贫困村提升工程公司、扶贫开发公司、林投、交投、水投等五个融资平台，撬动社会资本投向脱贫攻坚；市财政新增财力50%用于脱贫攻坚，各贫困县新增财力的主要部分也要倾斜用于脱贫攻坚，实现财政专项扶贫资金投入总量和增幅保持“双较大增长”。引导和鼓励金融机构创新金融产品和服务，完善扶贫贴息贷款政策，扩大覆盖面，力争新增扶贫小额贷款10亿元。扎实开展“扶贫领域作风建设年”活动，深入推进扶贫领域腐败和作风问题专项整治，筑牢扶贫资金管理使用的“高压线”。增强内生动力。大力选树、广泛宣传脱贫致富典型，持续开展“传承好家风、争当文明户”评选活动，弘扬脱贫攻坚正能量。推广岚县经验，选聘老干部、老教师和农技员，进村入户宣讲帮扶政策、开展技能培训，不断提升贫困群众自我发展能力。

　　各位代表，乡村要振兴，贫困一定要攻克！我们要持续精准发力，集中攻坚深度贫困县，打造脱贫特色县，巩固提升摘帽县，坚决扛起政治责任和时代责任，确保进度和质量，让脱贫成效得到群众认可、经得起历史检验！

　　（二）加快构建现代产业体系，着力推动高质量发展。

　　围绕打造能源革命排头兵，以壮士断臂的勇气，延长资源产业链条，培育非煤产业、非资源产业，努力改变传统产业多新兴产业少、低端产业多高端产业少、资源型产业多高附加值产业少、劳动密集型产业多资本科技密集型产业少的状况，构建多元发展、多极支撑的现代产业体系。

　　推动传统产业规模化集群化发展。加快煤电铝（镁）材一体化改革发展。围绕建设孝义、兴县2个百万吨级和柳林、交口2个50万吨级铝材加工基地，按照“铝土矿不出市、氧化铝不流失、原铝水不落地”的原则，推动兴县中润一期50万吨合金铝项目全线投产，争取启动二期50万吨合金铝项目；加快柳林森泽50万吨铝镁深加工、孝义信发110万吨轻质合金、交口伊电50万吨铝材等项目前期工作，力争年内开工建设。推动局域电网增容扩网，争取晋能大土河2号机组、京能吕临105万千瓦机组并网发电，建成孝义晋能2×35万千瓦发电机组。推动煤、铝、电企业相互参股，实现大用户直供电等政策落地；引导铝工业企业围绕赤泥综合利用、煤矸石提取氧化铝等进行技术突破，创建煤电铝（镁）材一体化循环经济试点。推动煤焦传统产业向化产品方向延伸。围绕孝义梧桐、交城夏家营、离柳矿区3个千万吨和汾阳三泉500万吨新型煤化工基地提档升级，稳定减少焦化产能，推动焦化企业向园区集中集聚，向煤化工产品高端化方向发展。大力引进高新科技、金融资本和优秀人才，加快建设孝义鹏飞40万吨费托合成蜡、90万吨超级悬浮床焦油加氢和孝义金岩45万吨乙二醇联产3亿立方LNG、孝义金达2×5亿立方LNG项目，建成投产孝义金岩250万吨、金达150万吨大型焦化项目，推进中阳福裕220万吨焦化项目复工复建，开工建设汾阳金塔山130万吨焦化项目。推动白酒产业发展壮大。加快建设全国最大的清香型白酒生产销售基地，以汾酒集团为主建设国家级白酒交易会展中心，以杏花村经济开发区为主建设国家级白酒质量检验检测中心。支持汾酒集团改革发展，通过租购并举，推动中汾酒城释放全部产能。支持汾阳华樽、青花瓷，方山老传统、良泉，文水女皇、宗酒、龙泉等中小微白酒企业发展壮大，力争全市白酒产量和销量增长20%以上。推动钢铁产业提档升级，推进中钢50万吨矿用支护材料加工、100万吨线材深加工和航电新能源磁粉芯项目建设，加快文水海威技术转型。列出重大技改和产业延伸项目清单，积极争取省级技术改造专项资金，市县配套专项资金，促进传统产业装备水平、产品质量和附加值逐步提高。

　　坚持把文化旅游产业培育成战略性支柱产业。依托丰富的旅游资源，努力建设全域旅游示范市。着力锻造黄河旅游板块，以临县碛口古镇、石楼黄河奇湾为重点，打造百里黄河旅游带。实施旅游公路率先突破，加快建设沿黄扶贫旅游公路及生态绿道项目，全面开工建设兴县至柳林军渡一期工程，建成黄河干线碛口试验段；加快建设离石—碛口、离石—千年—庞泉沟—北武当山等旅游公路，构建“城景通、景景通”的旅游公路网。全面提升景区品质，以“乘汾酒号列车、赏山口村庄”“吕梁—一个令人向往的地方”为主题，重点挖掘红色文化、廉政文化等旅游资源，持续打造“游吕梁英雄故里、看红色经典展馆”品牌。推动岚县、交城创建国家全域旅游示范县。加强与省文旅集团等旅游战略投资商对接，实施重点景区改造提升。推进交城卦山玄中寺英雄广场、方山北武当山、孝义三皇庙孝河湿地公园金龙山创建国家5A级景区，岚县白龙山、柳林抖气河、中阳柏洼山等景区争创4A级景区。加快建设完善游客服务中心、停车场、特色餐饮、宾馆等旅游配套设施，加快无线网络全覆盖，推进旅游“厕所革命”，以设施改善带动服务水平提升。建设“大数据＋旅游”平台，推行大宗旅游奖励、旅游惠民一卡通、淡旺季景区门票优惠等政策措施，拉动旅游消费快速上升。

　　大力培育新产业、新动能、新增长极。大数据产业，深入实施2018年行动计划，做大做强华为云服务大数据中心、吕梁军民研究院云计算中心两大品牌，大力发展数据平台经济，加快建设“数谷吕梁”，努力跨入全国大数据第一方阵。8月中旬前建成投运华为大数据中心，年底前建成投用交城中交网通西部数据中心一期工程，推动吕梁军民研究院云计算中心提升市场化运用水平，加快建设吕梁国家数据产业园、中阳区域大数据科技人才孵化区及孝义城市综合体等项目，筹建山西省大数据研究院吕梁分院。支持华为承接省直有关厅局及周边地市灾备中心项目，启动建设水利信息化平台和扶贫云、卫生云、旅游云、工业云等项目，提升“数谷吕梁·智赢未来”活动影响力和知名度。新能源产业，加快建设孝义汉能移动能源产业园区项目，确保一期20MW柔性太阳能电池组件和600MW太阳能组件制造项目投产运行，打造移动能源领跑者；加快建设文水晋能清洁能源“互联网+光伏制造业”、离石华富星辰100MW太阳能电池组件项目。新材料产业，重点建设文水水木新碳材1万吨/年锂电池负极材料、离石中磁尚善金属软磁粉芯等项目。装备制造业，重点建设交城利虎汽车用安全玻璃、离石新龙重工煤机装备技改等项目。节能环保产业，重点建设兴安化工30万吨赤泥综合利用、柳林森泽煤铝高白氢氧化铝产品、山西国峰煤电粉煤灰综合利用、岚县三鑫继亨20万吨矿渣棉综合利用二期等项目。开工建设晋西北建筑产业园区。现代服务业，发挥吕梁机场、太中银铁路、瓦日铁路等交通大动脉作用，规划启动建设新区航空港物流区、吕梁综合物流园区，开工建设太原铁路局吕梁综合服务项目。引进红星美凯龙等行业龙头企业，在新区发展商贸服务业。发展“互联网+”、智慧物流等衍生业态。发展监理业、评估业、咨询业等新业态。出台优惠政策，支持金融业、运输业和商贸流通业发展。

　　各位代表，在新时代的春天里，资源型地区经济转型已经全面上升为国家战略。我们将紧紧抓住政策机遇，巩固拓展经济快速发展的良好态势，加快培育新动能，持续培植新要素，不辱使命、义无反顾地推动转型，努力走出具有鲜明吕梁特色的转型之路！

　　（三）深入推进“转型项目建设年”，以大平台大招商大项目夯实转型发展基础。

　　加快开发区改革创新发展。继续推进开发区整合设立扩区，争取吕梁经济技术开发区和岚县、交口3个省级开发区年内获批，文水、交城2个省级开发区扩区获批。加快开发区体制机制改革，积极推进“三化”改革，探索“建管办分离”。复制推广山西转型综改示范区管理权限，切实向开发区授权放权。抓好开发区基础建设，已批开发区抓紧启动四至范围核定工作；按照“五规合一”要求，各开发区启动编制总体规划，加快完善基础设施建设，做好“九通一平”，推动项目尽快入区。实行“一地一策”，分类处置闲置土地，土地指标向开发区倾斜。布局好开发区产业项目，确保各开发区工业投资增长20%以上。吕梁经济技术开发区主要布局大数据产业，孝义开发区主要布局煤电铝材产业，汾阳杏花村开发区主要布局白酒产业，交城开发区主要布局煤化工和大数据产业，文水开发区主要布局新能源和装备制造业，兴县开发区主要布局煤电铝材深加工产业，岚县开发区主要布局冶金和制造业。方山、临县主要推进全域旅游文化建设。支持有条件的开发区以“区中园”模式建设高新技术产业园和国际产业合作园，努力在对外开放、招商引资、引领转型上有新的突破，积极构建内陆地区对外开放新高地。

　　加大招商引资力度。坚持把平台化、品牌化招商引资作为“一号工程”，举办2018年“数谷吕梁·智赢未来”论坛、第二届世界酒文化博览会、煤焦化产业发展推介会和第三届吕梁铝工业论坛四大平台招商活动，举办中国作家节。加强与全国性的行业协会、各种商会和学会等中介机构合作，举办各类招商引资引智活动。支持各县市区打造临县“枣儿红了”、岚县“土豆花开了”等特色招商平台。重新梳理完善土地、税收、资源等优惠政策，精心策划包装一批招商项目。紧盯东南沿海资本富集地和产业转移输出地，主动走出去，实施目的地招商、以企招商、专业招商、委托招商，不拘形式引商引智，吸引大型央企国企、世界500强企业、高新技术企业、知名品牌企业落户吕梁。

　　全力扩大转型项目投资。扎实推进传统产业升级改造、新兴产业、科技创新等6大领域、531个已确定的建设项目。精心储备和推进一批技术含量高、市场占有率高、拉动力强的项目。做深做实项目规划选址、用地预审、环评审批、核准等前期工作，掌握建设主动权。把项目落地作为关键环节，建立完善领导包联、并联审批、协调调度、市县联动、统计监测、监督考核等机制，扎实开展“集中开工月”“打击恶意阻工扰工”等专项行动，顺利推进项目建设。

　　着力打造“六最”营商环境。深入开展“‘六最’营商环境建设年”活动，全市域推开企业投资项目承诺制改革，推行“五统一”工作机制，加快建立以“政府服务承诺+企业信用承诺”为核心的审批管理模式。继续推进入企服务常态化，落实企业减负政策，减轻企业负担。深化“放管服效”改革，加大简政放权和审批服务力度，年内投用市级政务服务中心，争取企业开办时间减少一半，项目审批时间砍掉一半，政务服务一网办通，企业和群众办事只进一扇门，最多跑一次在吕梁逐步成为现实。凡是没有法律法规规定的证明一律取消，进一步激发市场活力和社会创造力。

　　（四）实施乡村振兴战略，推进农业农村优先发展。

　　按照“产业兴旺、生态宜居、乡风文明、治理有效、生活富裕”的总要求，聚焦乡村产业振兴、人才振兴、文化振兴、生态振兴和组织振兴，建立健全城乡融合发展体制机制和政策体系，加快推进乡村治理体系和治理能力现代化，努力实现吕梁农业全面转型升级、农村全面繁荣进步、农民全面富裕发展。

　　大力发展现代特色农业。建设沿黄4县红枣产业基地，实施红枣产业“保卫战”；加快建设汾阳、中阳核桃和兴县小杂粮、岚县马铃薯、石楼蜂蜜、方山中药材、交口食用菌、孝义设施蔬菜、文水畜产品等基地县，规划建设一批红枣小镇、核桃小镇、沙棘小镇、土豆小镇、有机杂粮小镇等特色产业旅游小镇，真正把“一县一业、一村一品”做大做强。加快创建全国农业标准化示范市，重点打造孝义、方山、岚县、文水4个标准化生产示范县和孝义、交城2个农产品质量安全县。着力培育特色农产品品牌，把红枣、核桃、土豆、小杂粮、沙棘、蜂蜜、香菇、清香白酒打造成具有吕梁全域品牌的知名产品。

　　加快一二三产融合发展。深化农业供给侧结构性改革，大力发展功能农业，推动全产业链发展。抓好农产品深加工，强化项目审批、建设占地、用水用电、税收减免等政策支持，培育一批前景好、规模大、辐射带动能力强的农产品加工企业，带动全市农产品加工总量达到150万吨，销售收入突破160亿元。加快发展休闲农业和乡村旅游，大力实施乡村旅游富民工程，开发农家乐、乡村客栈、民俗展演、农业观光园等乡村旅游项目，推进汾阳贾家庄、离石信义、兴县蔡家崖、交城庞泉沟等一批特色旅游小镇建设，争取创建50个乡村旅游景区、100个乡村旅游客栈，把乡村旅游打造成农民增收的新渠道。进一步完善物流体系，加快建设汾阳核桃与杂粮、岚县马铃薯、方山中药材、柳林和临县红枣等专业批发市场，支持供销、邮政及各类企业延伸发展线上线下相结合的电商服务网点，全力打通特色产品走向市场的物流通道，力争年内电商销售额达到5亿元以上。大力发展会展经济，在京津、沿海分区域组织特色农产品巡展推介活动，精心筹办吕梁市首届名特优功能食品展销暨招商引资会、首届吕梁核桃产业高峰论坛，协办好第八届国际沙棘协会大会，加快吕梁特色农产品“走出去”步伐。启动“五库两平台一基金”工程，打通农业生产各个环节之间的联系，全面振兴农业产业。

　　全面深化农村改革。完成农村土地确权登记颁证工作，深入落实承包地“三权分置”制度，力争流转土地面积110万亩以上。推进农村“三变”改革，探索以“公司+基地+集体经济+农户”“公司+合作社+农户”等多种方式，实现资源变资产、资金变股金、农民变股东。探索林长制改革，推进集体林地“三权分置”,开展集体林托管试点。铺开农村集体产权制度改革，推进集体经营性资产股份合作制改革，组建村级集体经济合作组织，培育资产性收益扶贫试点100个，争取所有行政村集体经济收入“破零”。

　　推动农村各项事业全面发展。扎实推进农村四好公路建设，年内新改建农村公路1500公里；抓好饮水安全建设，年内实施757处饮水安全工程；推进人口大镇、特色小城镇和旅游重镇集中供热、供气步伐，重点对柳林李家湾、中阳金罗、方山大武、临县碛口、文水胡兰、汾阳贾家庄等人口集中乡镇和旅游重镇实施集中供热；推进天然气管网向县城周边村、国省道沿线以及输气管道途经的乡村延伸，在具备条件的乡村实施集中供气。

　　加快建设美丽宜居乡村，开展农村人居环境整治三年行动，继续引深“十大特色镇”“百村示范、千村整治”创建活动；继续在全市范围内的高速公路、国道、省道和县乡道路沿线，推进以绿化、美化、净化为内容的“三道三化”专项整治行动；实施农村垃圾、污水、厕所粪污治理；抓好乡村规划，突出晋西民居风格和特色；推进传统村落、宜居村庄、绿色村庄、特色景观旅游名村申报保护工作，打造让群众看得见绿、望得见水、记得住乡愁的幸福家园和美丽宜居乡村！

　　（五）坚决打好重大风险化解、污染防治攻坚战，营造良好的发展环境和生态环境。

　　坚决打好防范重大风险攻坚战。稳步推进联盛集团破产重整，依法合规推进中诚一号融资风险化解，加快岚县、汾阳、离石、方山、交口农信社改制步伐，坚决守住不发生系统性区域性金融风险的底线。进一步引深打击恶意逃废债务、非法集资、网上集资专项行动，建立健全守信激励、失信惩戒制度，营造良好金融生态。严格规范和遏制政府举债行为，按照“分类管理、区别对待、逐步化解”的原则，摸清隐性债务底数，严控增量，消化存量。

　　坚定不移打赢污染防治攻坚战。充分运用“人防、技防、联防”，坚持“建、转、提、停、罚”综合施策，全面实施工业污染源深度治理、扬尘污染治理、农村面源污染治理、重点河流环境整治等生态环保八大治理工程，坚决完成中央、省环保督察反馈36个问题75项整改任务。持续推进吕梁市区集中供热全覆盖，供热面积在去年增加500万平米基础上，再新增230万平米，供热范围再新增21个周边村。在市区周边1300平方公里“一区三县”所辖区域内，推进81个村512万平米清洁采暖改造。加强市区燃煤燃放烟花爆竹管控、大型机动车辆限行、扬尘污染整治，推动市区环境空气质量持续保持在全省重点城市前列。开工建设市区生活垃圾焚烧发电厂、餐厨垃圾处理厂、第二污水厂二期及污水收集管网工程，切实解决区域污染严重问题。以汾阳、文水、交城、孝义为重点区域，实施大气污染防治联防联控措施，推动城区环境空气质量明显好转。整治市区刘家湾沟、枣林沟、北川河一号坝至交口大桥段等黑臭水体，市区建成区率先消除黑臭水体。完成汾阳东二环西侧黑臭水体整治，其他县市黑臭水体消除比例达到80%以上。继续推进畜禽养殖粪污治理，完成90户规模养殖场粪污处理设施建设任务。推进净土行动，开展土壤污染状况详查，实施农用地土壤环境分类管理，推进土壤污染治理与修复。

　　全力推进生态修复治理。认真落实河长制，制定市管河流“一河一策”方案，开展河流水质改善专项整治，推进文峪河、三川河、磁窑河等重点河流水污染防治，全面禁止垃圾入河，严格控制污水入河，推动横泉水库饮用水源地保护，实现国省控断面水质达标。突出抓好黄河、汾河吕梁段和吕梁山生态重大保护修复工程。开工建设文水、孝义等沿汾县（市）堤外湿地项目，打造黄河生态经济带、汾河生态修复经济带和吕梁山国家公园“两带一园”。交口县创建森林示范县，每个县创建1—2个森林小镇，全市创建40个生态文化村。合理划定生态保护红线，推进绿色发展考核评价，着力解决环境突出问题，让我们的天更蓝、山更绿、水更清、环境更优美，让“吕梁蓝”成为我们的新名片！

　　（六）坚持以改革创新引领发展，不断解放和发展社会生产力。

　　发展前进一步，改革创新就要前进一步。我们要把改革的突破点推进到离市场、离群众最近的地方。

　　深化能源供给侧结构性改革。提高煤炭供给体系质量。坚定走“减、优、绿”之路，坚持淘汰落后低效产能与大力发展先进产能相结合，推进交口华瑞煤业与孟家焉、蔡家沟和梁家沟3座煤矿减量重组，退出煤炭产能60万吨、减量置换61.8万吨。加快安全高效矿井建设，年内竣工投产矿井10座，新增安全高效矿井5座、先进产能600万吨，安全高效矿井比重达到80%以上。调整优化能源产业结构。加强电力需求侧管理，推进电力直接交易，力争全年直供电量达到28亿度。实施煤层气发电提速、风电壮大提升工程，年内建成柳林华晋焦煤8兆瓦、文水金地赤峪24兆瓦瓦斯发电项目，加快建设华电汾阳杨家庄10万千瓦、交口棋盘山10万千瓦、国电投河口二期50兆瓦等风电项目。

　　深化基础性关键领域改革。稳步推进市县国有企业改革。按照“分级管理、统筹推进”原则，通过股权融资、股权合作，吸引优质资本参与，加快推进离柳集团股权多元化改革、吕梁中药厂专业化改革重组。统筹解决好山西第四纺织机械厂、吕梁永固制砖公司和吕梁药材公司等停产停业企业的历史遗留问题；全面完成国有企业“三供一业”及教育、医疗、社区等公共服务机构的分离移交工作，有效化解国企历史包袱。推进民营企业发展壮大，坚持“两个毫不动摇”，加强合法产权保护，支持民营经济发展。继续实施民营企业“三个一”提升工程，组织培训100名企业家、1000名经营者、10000名技术骨干，提升企业整体素质。大力支持个转企、小升规、规改股、股上市，加大考核力度，年内新创办小微企业4000户，新发展“小升规”企业30户，每县至少完成1户企业股份制改造，争取全市挂牌上市3户企业。

　　深化投融资体制改革。创新运用基础设施和产业发展基金支撑转型。充分发挥市国投、交投、水投、林投等投资功能作用，运用好去年设立的基础设施基金，推进市政设施、道路交通、水利等基础设施建设。积极推广“政府+企业”“政府+金融机构”“政府+企业+金融机构”“企业+金融机构”等基金模式，发挥好中睿、铝基新材料等6支产业基金作用，撬动社会资本投向新兴产业发展；引导支持民间资本联合起来设立投资基金，鼓励发行企业债券，推动盘活存量、融通资金，推动转型升级。进一步推广PPP模式，举办PPP项目推介会，推进政府与社会资本合作。推动祁离高速等30个PPP项目加快建设，重点培育一批基础设施、脱贫攻坚、公共服务示范项目，形成可复制推广的典型经验。

　　推进科技创新和人才支撑。继续推进以科技创新为核心的全面创新,深化与签约院校单位的合作，重点支持北航中汇科技孵化器、中钢—北航技术研发中心建设省级科技孵化器和技术研究中心，加快推进吕梁国家农业科技园区建设。深入开展“百家院校科技成果走基层走进吕梁活动”，搭建与国内外先进科技成果有效对接的桥梁和纽带。加快离石、孝义、交城省级双创示范基地建设，争取中阳列入全省第二批双创示范基地，年内建成省级及以上工程技术研究中心8家、科技企业孵化器3家、众创空间11家。鼓励企业建设“双创”基地和服务平台，支持建立职工创新工作室，推动大众创业、万众创新深入发展。继续实施“百千万人才工程”和“百校千人计划”，全面落实我市《开发区引进高层次科技人才实施意见》，建立科技人才引进专项资金，对引进各类科研项目和高层次人才进行奖补，对获得国家、省级、市级科学技术进步奖的个人和单位给予专项奖励；围绕县域主导产业发展技术需求，支持县（市、区）建立院士工作站或博士后工作站。

　　各位代表，惟改革者进，惟创新者强，惟改革创新者胜。我们要坚定不移地走改革创新之路，只要是为了人民利益的改革，我们万难不辞、万险不避！

　　（七）加快推进城镇基础设施建设，着力提升城镇化水平。

　　推动城市建设提档升级。坚持主城区、新城区一体化发展，统筹规划建设宜居城市。持续实施“双十”重点工程，完善基础设施，补齐发展短板，提高城市综合承载能力。主城区建设，启动实施市区雨污分流管网改造、东川河步道、东城区供水等10个重点项目。新区建设，完成吕梁医疗卫生园区、吕梁广场、如意湖等工程建设，推动新区路网、两山绿化、吕梁高等师范专科学校、吕梁一中、吕梁三中、规划馆、群艺馆和七、八、九号湖10个重点项目建设。积极推进盛地大道、广电大楼等项目前期工作。市场化引进碧桂园、富力城，发展房地产业，建设城市综合体。市区公共自行车在去年投放2000辆的基础上再增加1000辆。积极推进柳林撤县设区，拓展市区发展空间。进一步理顺市区两级土地、环卫、公安等管理执法体制，建立科学有效的城市管理体制机制。加快推进智慧城市、海绵城市建设，着力提升城市品质。

　　持续开展“五城联创”活动。交城、汾阳、交口3县（市）启动创建国家卫生城市，市区启动创建国家园林城市，汾阳、岚县、交口启动创建国家园林县城，交城启动创建省级园林县城和省级文明城市，争取孝义梧桐、临县碛口申报国家级特色小城镇，中阳枝柯、文水开栅、交口双池申报省级特色小城镇。统筹城市地上地下、“面子里子”工程，加快推进污水处理、垃圾处理、供气等公共服务设施建设，进一步提高城市公共服务水平和综合承载能力。

　　加快城镇基础设施建设。积极做好太原—吕梁—延安高铁前期工作，加快推进吕梁航空口岸临时开放。加快建设静兴和祁离高速公路，争取开工建设离石至隰县西纵高速和国道209市区段改线、新国道太克线三期工程，启动建设大武互通立交工程；做好汾阳至石楼高速公路前期工作。实施省道汾柳线改造工程、省道248三交—大宁公路改建工程。加快推进12处县域小水网分水口和25座调蓄水库前期工作，争取开工建设一批调蓄水库。加快峪口沟水库前期工作进度，争取尽早立项。加快孝义500千伏魏都输变电工程前期工作，开工交口、石州220千伏等输变电工程。

　　（八）持续保障和改善民生，让改革发展成果更多惠及人民群众。

　　大幅提升教育能力和水平。首先要办好市区教育，新建小学10所、初中3所、高中1所，深化市区贺昌中学等8所公办中学体制机制改革。全面实施“学前教育第三期行动计划”，新建公办幼儿园29所、普惠幼儿园31所，每个乡镇都建成一所中心幼儿园。提高幼儿教师待遇，学前教育公办经费列入财政预算。推进义务教育优质均衡发展，优化农村学校布局，办好寄宿制学校。实施“高中普及攻坚计划”，加快推进普通高中新课程改革。加大县级职教中心建设力度，13个县（市、区）全部建成职教中心。支持和规范民办教育发展，办好特殊教育。实施校长、教师培训提升工程，加强师德师风建设，提高教师待遇，打造一支人民满意的教师队伍。开展最美乡村教师评选表彰工作，营造浓厚的尊师重教氛围。

　　加快卫生健康事业发展。推进医疗、医保、医药“三医联动”，全面深化县乡医疗卫生机构一体化改革,加快理顺县级公立医疗机构管理体制，推动分级诊疗取得更大进展。年内建成投运市人民医院和汾阳医院医技综合大楼，完成市中医院改造。启动吕梁卫生健康职业学院建设前期工作。继续开展改善医疗服务行动计划，县级医疗集团全部与三甲医院建立医联体，基层医疗卫生服务机构达标率达到95%以上，让老百姓在家门口就能享受到优质的基本医疗服务。加强疾病防控、妇幼保健和爱国卫生工作。推进农村卫生厕所改造工作。完善全面两孩配套政策，为3万名城乡怀孕妇女提供免费产前筛查和产前诊断服务。

　　全面发展社会事业。落实积极就业政策，实施全民技能提升工程，做好高校毕业生、农村转移劳动力、城镇就业困难人员、军队退役人员和残疾人等重点群体就业工作。加强社会保障，实施全民参保计划，参保率达到95%以上。积极推广医养结合，大力发展养老服务业，完成90个农村老年人日间照料中心建设任务。全力推动吕梁特仪工程开工建设，市社会福利院主体竣工，市儿童福利院具备运行条件，市老年公寓投入运行。启动三年棚户区改造计划，完成棚户区改造7985套。全面落实最低工资标准、提高公职人员工资和离退休人员待遇、责任制考核奖励等政策，千方百计增加居民收入。

　　提升公共文化服务水平。建成吕梁图书馆。广泛开展群众性文化体育竞技活动，继续推进文化科技卫生“三下乡”、农村公益电影放映、免费送戏下乡惠民演出等文化惠民工程。加强与中央音乐学院深度合作，推动我市文化艺术事业加快发展。深入挖掘地方特色文化资源，推出一批精品力作。维修老城区体育馆，加快新区体育中心建设，做好第二届青运会吕梁赛区筹备工作。推进全民健身运动。

　　做好安全稳定等工作。牢固树立安全发展理念，以“四铁”要求推动全市安全生产形势持续稳定好转。扎实推进安全生产领域改革发展，建立健全安全生产责任体系、监管执法体系、预防控制体系和应急救援体系，压实各级党委政府的领导责任、行业领域的监管责任和企业的主体责任，坚决预防和遏制重特大安全事故发生。深化煤矿、道路交通、危险化学品、非煤矿山、冶金工贸等重点行业领域专项整治。按照“一张网、一张图、一张表、一盘棋”的要求,完成安全生产综合监管信息化平台建设。有序推进煤矿、非煤矿山、危险化学品等高危行业强制实施安全生产责任保险制度的落实，积极开展安全教育基地、安全文化示范企业创建活动，提升本质安全水平。深入开展食品药品重点领域薄弱环节专项治理，完善从生产到消费全链条监管，保证“舌尖上的安全”。健全立体化社会治安防控体系，试点建设“雪亮”工程，加快建设公安数据中心、合成作战平台、视频监控综合管理服务平台。深入开展扫黑除恶专项斗争，积极防范和依法严惩各类违法犯罪行为。严格执行重大事项、重点工程、重大决策社会稳定风险评估制度，从源头上、根本上预防和化解社会矛盾，确保人民生命财产安全、社会和谐稳定。重视国防教育、人防和兵役工作；加强统计、气象、文物保护、抗震设防、民族宗教、档案方志等工作。支持各民主党派和各人民团体工作。

　　各位代表，久久为功，功成有日。我们要静心用力，坚持不懈地一年接着一年干，一件接着一件办，锲而不舍兑现各项民生承诺，让吕梁人民为工作生活在吕梁而自豪，让吕梁人民生活随着经济发展一年更比一年好！

　　三、扎实推进全面从严治党和政府自身建设

　　各位代表，进入新时代，政府工作要有新气象新作为。我们一定不忘初心，牢记使命，按照习总书记提出的增强“八个本领”、锤炼“五个过硬”的要求，抓好推进全面从严治党，切实加强政府自身建设，努力当好新时代的“答卷人”。

　　全面加强党的政治建设。坚持和加强党的全面领导，坚持党要管党、全面从严治党。牢固树立“四个意识”，增强“四个自信”，坚定维护以习近平同志为核心的党中央权威和集中统一领导，自觉维护习近平总书记党中央的核心、全党的核心地位，自觉把维护以习近平同志为核心的党中央权威和集中统一领导作为最高政治原则和根本政治规矩，坚决执行党的政治路线，严格遵守政治纪律和政治规矩，坚决在政治立场、政治方向、政治原则、政治道路上同以习近平同志为核心的党中央保持高度一致。带头学习宣传贯彻习近平新时代中国特色社会主义思想和党的十九大精神，努力在学懂弄通做实上下真功夫、实功夫，在学习领会上先学一步、学深一层，努力带头学出觉悟、学出信仰、学出担当。坚持三个“一以贯之”，扎实开展“不忘初心，牢记使命”主题教育，不折不扣地把党中央和省委、市委各项决策部署落到实处。

　　全面推进法治政府建设。坚决维护宪法权威，捍卫宪法尊严，保证宪法实施。严格规范公正文明执法，做到有权不可任性，用权必受监督。认真执行市人大及其常委会的决议决定，自觉接受人大和政协监督。认真办理市人大代表建议和市政协委员提案。坚持科学民主依法决策，大力推进依法行政，落实政府法律顾问制度，推动政府工作全面纳入法治轨道。政府带头讲诚信，依法履行合同约定，带动社会诚信建设，让诚信成为吕梁的靓丽名片。

　　全面加强党风廉政建设。认真落实全面从严治党的主体责任，严格规范党内政治生活，推动政府系统党风廉政建设向纵深发展。严格落实中央“八项规定”和实施细则精神，驰而不息纠正“四风”，重点整治形式主义、官僚主义新表现。紧盯重点领域和关键环节，深化廉政风险防控。加强审计监督，突出抓好巡察发现问题整改落实。认真落实省委“五倡导、五反对”要求，坚决纠正表态多调门高、行动少落实差等问题，大力整治庸政懒政怠政和不作为、乱作为。认真落实市委激励干部担当作为干事创业办法、支持干部改革创新合理容错办法，为敢于担当者担当，对敢于负责者负责，推动广大干部放开手脚干事创业，干出实打实的新业绩。

　　全面加强效能建设。深入开展“工作落实年”活动，大力推行重点工作微信群制度，健全重点工作清单制度和责任分解机制，加大“13710”督查督办力度,完善问责和激励机制，确保每一项工作、每一条措施都落到实处。各级政府工作人员一定要扑下身子抓落实，冲锋在前、苦干实干，以“钉钉子”精神真正把各项工作落细落小落实。大力弘扬“吕梁精神”，在脱贫攻坚和转型发展主战场上，锻造一支敢担当、善作为、作风硬的干部队伍，奋力谱写新时期“吕梁英雄传”。

　　各位代表，幸福都是奋斗出来的，蓝图都是实干实现的。让我们更加紧密地团结在以习近平同志为核心的党中央周围，在市委的坚强领导下，凝心聚力、奋力攻坚，为决胜全面建成小康社会、开拓转型综改新局面、开启新时代吕梁发展新征程而努力奋斗!
